# Ultrastructural deposits appearing as “zebra bodies” in renal biopsy: Fabry disease?– comparative case reports

**DOI:** 10.1186/s12882-017-0571-0

**Published:** 2017-05-12

**Authors:** Precil Diego Miranda de Menezes Neves, Juliana Reis Machado, Fabiano Bichuette Custódio, Maria Luíza Gonçalves dos Reis Monteiro, Shigueo Iwamoto, Marlene Freire, Marisa França Ferreira, Marlene Antônia dos Reis

**Affiliations:** 1 Nephropathology Service, Federal University of Triângulo Mineiro, Praça Manoel Terra, 330, Uberaba, MG CEP: 38015-050 Brazil; 2 Renal Therapy Unit, Federal University of Triângulo Mineiro, Praça Manoel Terra, 330, Uberaba, MG CEP: 38015-050 Brazil; 3 Rheumatology Service, Federal University of Triângulo Mineiro, Praça Manoel Terra, 330, Uberaba, MG CEP: 38015-050 Brazil; 4 Nephrology Service, Felício Rocho Hospital, Rua Uberaba, 500, Belo Horizonte, MG CEP: 30180-080 Brazil

**Keywords:** Fabry Disease, Hydroxychloroquine, Lysosomal Storage Disorder, Renal Biopsy, Systemic Lupus Erythematosus

## Abstract

**Background:**

Fabry Disease (FD) is a genetic disorder caused by alpha-galactosidase A deficiency. Certain drugs, such as hydroxychloroquine, can produce renal deposits that mimic morphological findings seen in FD, characterizing a type of drug-induced renal phospholipidosis.

**Case presentation:**

Case 1: A 28-year-old female patient with systemic lupus erythematosus who had been using hydroxychloroquine for 14 months presented subnephrotic proteinuria. Renal biopsy showed deposits compatible with FD. Neither activity analysis of alpha-galactosidase A nor genetic analysis were available and were not performed. These deposits were not detected in a subsequent renal biopsy three years after withdrawal of the medication, characterizing a possible hydroxychloroquine-induced renal phospholipidosis. Case 2: A 29-year-old male patient presented with acroparesthesia, angiokeratomas, cornea verticillata and subnephrotic proteinuria. Deposits compatible with FD were detected upon renal biopsy. The evaluation of alpha-galactosidase A showed no activity in both blood and leukocytes. Genetic analysis identified an M284 T mutation in exon 6, and such mutation was also found in other family members.

**Conclusion:**

Clinical investigation is necessary in suspected cases of Fabry Disease upon renal biopsy in order to confirm diagnosis. Drug-induced renal phospholipidosis should be considered in differential diagnosis in cases with intracellular osmiophilic, lamellar inclusions in electron microscopy.

## Background

Fabry Disease (FD) is an X-linked inborn error of metabolism caused by deficiency of the lysosomal enzyme alpha-galactosidase A that causes a systemic intracellular accumulation of glycosphingolipids, especially globotriaosylceramide (GL-3), thus leading to cellular dysfunction [1–4]. Clinical manifestations include ophthalmological signs (corneal dystrophy), skin manifestations (angiokeratomas), neurological anomalies, renal and cardiovascular diseases [1, 5, 6].

Although it is a X-linked disease, female heterozygotes may develop the disease and previous studies even showed more severe visceral involvement in female than in male patients [1, 3, 5, 6].

Early stages of renal damage in FD is characterized by disorders of urine concentration and eventually proteinuria, the most common sign observed at the diagnosis and that may be associated with nephrotic syndrome [2]. Evaluation of renal biopsy shows GL-3 deposits in several cells such as podocytes, glomerular endothelial cells, mesangial cells, tubular epithelial cells and vascular endothelial cells. In many cells, cytoplasm vacuolization can be identified by light microscopy. Ultrastructurally, intracellular osmiophilic structures appearing as “lamellar bodies” or “zebra bodies” invariably indicate the disease [1, 7–9]. As previous described in the literature, phospholipidosis induced by drugs such as amiodarone, chloroquine and hydroxychloroquine may cause histological changes that mimic typical FD findings [8–11]. Drug-induced phospholipidosis consist in a challenging differential diagnosis for pathologists.

Two cases are reported herein: one patient with systemic lupus erythematosus (SLE) presenting a possible hydroxychloroquine-induced renal phospholipidosis mimicking FD and another patient with confirmed FD. The clinical findings and histopathological features of the two cases were compared. To the best of our knowledge, this is the first case report to document on electron microscopy the complete resolution of “zebra bodies” noted in the first biopsy after withdrawal of the drug.

Authors declare that adhered to the CARE guidelines for case reports.

## Case presentation

### Case 1

A 28-year-old female patient, previously hypertensive, was diagnosed with systemic lupus erythematosus (SLE) 14 months earlier, and since then was in treatment with captopril, hydrochlorothiazide, prednisone and hydroxychloroquine at 400 mg/day. She had daily fever (39 °C) for three months, as well as generalized erythema, photosensitivity and asthenia, clinical findings of disease activity. Laboratorial tests were as follows: Blood Urea Nitrogen (BUN): 8.87 mg/dl (reference range: 6–21 mg/dl); serum creatinine: 0.8 mg/dl (reference range: 0.4–1.1 mg/dl); estimated glomerular filtration rate (eGFR) measured using CKD-EPI equation: 116 ml/min/1.73m^2^ (reference range: >90 ml/min/1.73m^2^); positive anti-nuclear antibody test (HEp2): 1/160 (reference: negative); and positive Antinuclear Antibody (ANA): 1/640 (reference: negative). Other causes of proteinuria were excluded. Kidneys were unremarkable on ultrasound examination. Analysis of urine revealed a 24-h proteinuria of 600 mg (reference: no proteinuria) with no signs of hematuria or leukocyturia. The patient was not tested for erythrocyte dysmorphism or for lipid-laden epithelial cells due to unavailability of such tests at the referred service. Renal biopsy was performed for evaluation of lupus nephritis. There was no family history of Fabry Disease.

**Fig. 1:**
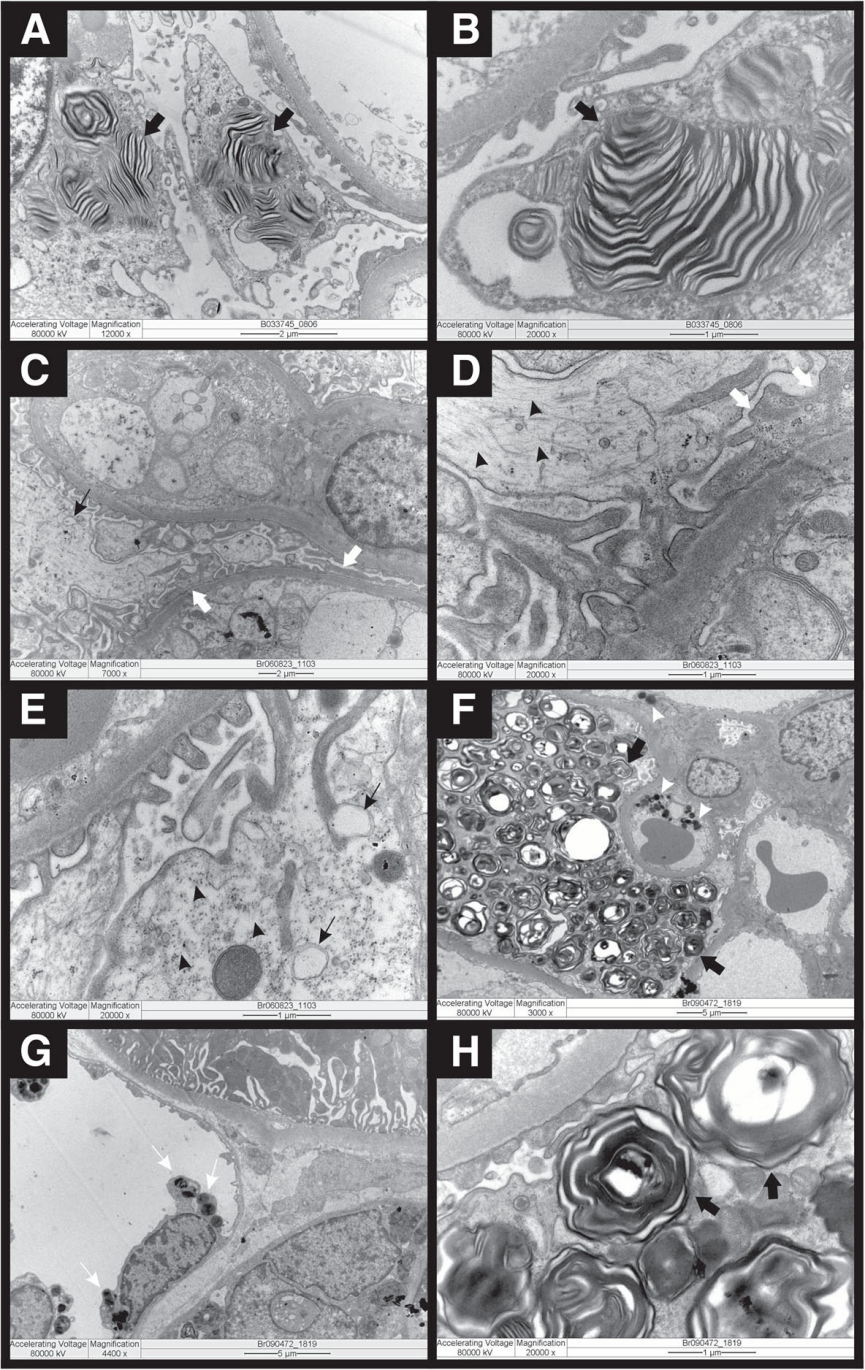
Electron microscopy: Case 1 - first biopsy (**a** and **b**): intralysosomal, osmiophilic, lamellated and sometimes concentric inclusions in the cytoplasm of podocytes (*black arrows*) resembling or “zebra bodies”. Case 1 – second biopsy (**c**, **d** and **e**): podocytes presented cytoplasmatic swelling (*arrow heads*), sometimes forming optically empty vacuoles (*slim black arrows*) and there are some podocyte foot process effacement (*white arrows*). The lipid deposits seen in the previous biopsy were no longer detected. Case 2 - (**f**, **g** and **h**): deposits of lamellate, lipid-like, electron-dense material forming concentric bodies, “zebra bodies” in the cytoplasm of podocytes (*black arrows*) (**f**, **h**) and endothelial cells in both glomerular (*white arrow head*) (**f**) and peritubular capillaries (*white slim arrows*) (**g**).Image magnifications are specified in each lower border

Renal biopsy found: I) Light microscopy: eight glomeruli with mild hypercellularity in some mesangial regions. Tubular, interstitial and vascular compartments were normal. II) Immunofluorescence: positive granular mesangial anti-IgM in glomeruli and mildly positive anti-C3 in vascular wall. III) Electron microscopy: intralysosomal, osmiophilic, lamellar and sometimes concentric inclusions in podocytes resembling “zebra bodies” (Fig. 1: a and b) – a finding compatible with both FD and drug-induced phospholipidosis. Given the presence of active SLE, hydroxychloroquine was replaced by thalidomide at 100 mg/day. The patient had normal renal function and there was no evidence of angiokeratomas, cornea verticillata, peripheral sensory neuropathy or other clinical or laboratory features consistent with Fabry disease, even after extensive investigation. The activity level of alpha-galactosidase A was not performed at that time (2003) due to technical problems with the blood sample. Genetic evaluation was not widely available at that time and it was not performed either. After an indefinite period of replacement of hydroxychloroquine by thalidomide and having increased captopril dose, proteinuria was reduced to undetectable levels.

A second renal biopsy was performed three years later (2006) due to new onset of subnephrotic proteinuria (24-h proteinuria of 500 mg; reference: no proteinuria) without hematuria/leukocyturia and with normal renal function. Light microscopy: four glomeruli, one of them globally sclerotic; mild mesangial hypercellularity and mild foci of interstitial fibrosis. Immunofluorescence: Anti-IgG weakly positive in some mesangial regions. Mesangial weakly positive anti-IgM and anti-kappa in two glomeruli. Electron microscopy: podocytes with cytoplasmic swelling, sometimes forming optically empty vacuoles and foot process effacement. Lipid deposits found in previous biopsy were no longer observed (Fig. 1: c, d and e). the findings were compatible with class 1 minimal mesangial lupus nephritis. The fact that the treatment with hydroxychloroquine had been interrupted added to the lack of clinical/laboratory evidence of FD, lead to the conclusion that the deposits detected in the first biopsy were most probably due to hydroxychloroquine-induced renal phospholipidosis.

### Case 2

A 29-year-old man presented with arthralgia, pain and paresthesia in fingers. Hypertension, diabetes mellitus, gross hematuria or photosensitivity were absent. There was no family history of Fabry Disease. Upon physical examination, he was normotensive and there were no signs of arthritis or edema. Laboratory tests showed: BUN: 10 mg/dl (reference range: 8–24 mg/dl); serum creatinine: 0.8 mg/dl (reference range: 0.5–1.2 mg/dl); estimated glomerular filtration rate (eGFR) measured using CKD-EPI equation: 120 ml/min/1.73m^2^ (reference range: > 90 ml/min/1.73m^2^); 24-h proteinuria: 1.9 g (reference range: no proteinuria); urine examination without hematuria or leukocyturia; normal serum complement and normal serum protein electrophoresis. Investigations for other causes of proteinuria were negative. Renal ultrasound was normal. Given these findings, treatment with Enalapril (5 mg/day) was initiated. The patient continued to report pain in the right ankle associated with pain and paresthesia in hands. Diagnosis of FD was suggested. Examination revealed cornea verticillata and angiokeratomas on the back. Echocardiogram showed endocardial border binary appearance and posterior mitral leaflet prolapse, with left ventricular wall thickness of 0.8 cm (normal range: 0,7–1.2 cm), left ventricular mass of 65 g/m^2^ (normal range: 57-75 g/m^2^) and no signal of systolic nor diastolic dysfunction. Activity of alpha-galactosidase A, which can be measured in plasma, leukocytes or cultured fibroblasts as well as from samples of whole blood on filter paper, was not detected in filter paper or leukocytes, thus confirming the diagnosis of FD.

Renal biopsy showed fine cytoplasmic vacuolization of podocytes; in immunofluorescence, sample consisted of connective tissue without reaching kidney and in electron microscopy there were electron-dense lamellate lipid-like deposits forming concentric bodies, “zebra bodies” in the cytoplasm of podocytes and endothelial cells in both glomerular and peritubular capillaries, which is compatible with FD (Fig. 1: f, g and h).

Clinical manifestations associated with the absence of detectable alpha-galactosidase A activity in samples collected on filter paper (normal range > 2.5 μmol/L/h) and in leukocytes (normal range > 1.4 nmol/mg protein/h) and with renal biopsy findings, led to the diagnosis of FD. Upon genetic analysis, M284 T mutation in exon 6 of GLA gene was detected in the patient as well as in her mother and sister, who were both asymptomatic. Enzyme replacement therapy was initiated, as it can improve symptoms due to clearance of lysosome deposits [12] and according to expert recommendation to initiate it as soon as clinical signs of the disease are detected in kidney, heart or brain in classically affected males [13]. Enalapril dose was increased to 20 mg/day. Proteinuria levels were reduced to approximately 500 mg/24 h.

## Discussion

In this paper, two cases of renal biopsy showing identical lipid deposits were presented. In the first case, these lipid deposits were due to inhibition of intralysosomal alpha-galactosidase A activity (presumable hydroxychloroquine-induced renal phospholipidosis) and in the second case, due to a mutation in the gene encoding the referred enzyme.

FD is a X-chromosome linked genetic disorder characterized by disturbance in glycosphingolipid catabolism, caused by a deficiency of the enzyme alpha-galactosidase A. Early signs and symptoms occur from childhood to adolescence and include intermittent paresthesia and acroparesthesia, “Fabry crisis” (episodes of intense pain), recurrent fever, angiokeratomas, cornea verticillata, mild proteinuria, globotriaosylceramide in urinary sediment and digestive symptoms such as both diarrhea and constipation, nausea, vomiting and abdominal cramps. Manifestations in adolescence and adulthood include renal disorders (which can progress to end-stage kidney disease) and hypertension, cardiovascular changes (myocardial infarction, cardiac hypertrophy, valvular abnormalities and arrhythmias), cerebrovascular complications (risk of stroke) and pulmonary complications (respiratory obstruction and dyspnea). Late manifestations (renal, cardiac, and neurological) are the most severe complications and are the cause of death and reduced life expectancy in patients with FD [1, 4, 6, 14].

Clinical manifestations of both reported cases were compared. The patient with renal phospholipidosis showed no typical clinical signs that supported the hypothesis of FD. In contrast, the patient with FD had arthralgia, acroparesthesia, angiokeratoma and cornea verticillata, manifestations that, when combined, are highly suggestive of FD. The diagnosis was confirmed by the lack of plasma/leukocyte alpha-galactosidase A activity, as well as by genetic mutation analysis, with an M284 T mutation in exon 6.

Diagnosis of FD in male individuals can be performed by measuring enzyme activity in peripheral blood (screening/initial diagnosis) or in leukocytes, and electron microscopy findings are adjunct for diagnosis. Analysis of enzyme activity in blood and leukocytes may be normal in women, then evaluation of genetic mutation is essential for diagnosis [1, 6, 15]. In phospholipidosis case in a female patient, neither enzyme activity nor genetic mutation analysis for FD were performed due to a lack of access to such examinations (2003). The diagnosis of drug-induced renal phospholipidosis was made based in the fact that the deposits were no longer evidenced in the second biopsy (2006) after withdrawal of hydroxychloroquine. In the second case, of the male patient, not only enzyme activity was negative, but it was also possible to perform genetic evaluation with identification of an identical mutation in the patient and in his mother and sister.

Furthermore, recent studies [1, 4, 15] have focused on microscopic examination of urine as a key tool for the diagnosis of FD through vacuolated epithelial cell slides showing GL-3 deposits, which resemble a Maltese cross under polarized light. In their study, Selvarajah et al. [15] showed that such cells had a sensitivity and specificity of 100% in patients with FD and they were present regardless of gender, albuminuria, renal function or usage of enzyme replacement therapy. The excretion of such particles was further associated with urinary levels of albumin. This examination could be used as an additional tool for the diagnosis of FD in women since enzyme activity levels may vary. Evaluation of positive anti-CD77 urinary cells (GL-3 accumulation marker) had a sensitivity of 97% and specificity of 100% in patients with FD, but it is more expensive than Maltese cross detection through polarized light microscopy. Urine evaluation under polarized light microscopy was not performed in these cases at the time, as it was not part of the service routine examination.

Renal involvement in FD affects mainly visceral epithelial cells (podocytes) whose cytoplasm becomes finely vacuolated, characterizing lipid accumulation. Parietal epithelial cells, mesangial cells and vascular myocytes may also be affected [7–10, 15]. Lipid deposits can be identified by light microscopy in glutaraldehyde-fixed and plastic embedded ultra-thin sections (0.5 μm) stained with toluidine blue or methylene blue [8]. Electron microscopy revealed enlarged lysosomes containing multiple electron-dense bodies appearing as lamellar bodies or zebra bodies, findings that characterize the lipid nature of deposits. In a manuscript proposing a classification score for renal biopsies of patients with FD, Fogo et al. [7] showed deposits are present even before clinical presentation. Moreover, several degrees of glomerulosclerosis and interstitial fibrosis can also be observed in individuals with minimal or even absent proteinuria, and these are the histological findings that better correlate with renal disease progression [7].

Drug-induced phospholipidosis is a term referring to intracellular accumulation of phospholipids and lamellar bodies, which are also microscopic indicators of lipid storage disorders [8–11]. Since alpha-galactosidase A is involved in both diseases, they have similar light and electron microscopic features. This fact may result in misdiagnosis, especially when patient history is not available for pathologists [9]. In case 1, the second biopsy showed no renal lesions after discontinuation of hydroxychloroquine. This fact, together with the absence of clinical manifestations of Fabry disease, points to the diagnosis of hydroxychloroquine-induced renal phospholipidosis.

Lipid deposits seen in both FD and drug-induced renal phospholipidosis are found in lysosomes, cellular organelles containing several hydrolytic enzymes such as lipases, phospholipases and proteases. Cationic amphiphilic drugs as chloroquine and amiodarone have hydrophilic ring and hydrophobic region, a feature that enables these drugs to readily pass through lysosomal membrane. After crossing this membrane, chloroquine, particularly – which is a weak base – is protonated in low pH medium and remains enclosed within the organelle because it cannot cross membrane hydrophobic layer. This chloroquine accumulation leads to inhibition of phospholipases A and C and alpha-galactosidase A, as well as to consequent blockade of intralysosomal phospholipid catabolism, which culminates in formation of deposits identical to those seen in FD [8–10]. Chloroquine and hydroxychloroquine have a similar chemical structure, thus suggesting the triggering mechanism of renal phospholipidosis is similar in both drugs [8, 10].

Lipidosis induction by drugs is dose dependent and reversible after discontinuation of their usage [16] but in humans, there are rare information regarding both the reversion rate [17] and the dose needed to induce phospholipidosis, probably due to the paucity of reports and diagnosis challenges. In a paper that reported hydroxychloroquine renal toxicity, there was increasing proteinuria, creatinine clearance of 67.2 mL/min (1.12 mL/s) and serum creatinine level of 1.1 mg/dL (97 mol/L). After six months of drug discontinuation, a significant reduction of proteinuria levels (from 3.38 g/24 h to 2.16 g/24 h) was observed but serum creatinine level persisted at 1.1 mg/dL (97 mol/L) with decreased creatinine clearance 58 mL/min (0.97 mL/s) [9]. In addition, there is a report of hydroxychloroquine prolonged use due to systemic lupus erythematosus with creatinine-clearance of 64 ml/min and 2 g/day proteinuria, in which 15 months after renal biopsy diagnosis, proteinuria levels ranged between 1.8 and 5 g/day and creatinine clearance remained stable [11]. In our case, the exactly time course for achieving undetectable levels of proteinuria after drug withdrawal was not known. The drug was used during 14 months in a dose of 400 mg/day, with a cumulative dose of 168 g. A case report of a Sjogren’s syndrome female patient who developed hydroxychloroquine-induced kidney disease refers a cumulative dose of hydroxychloroquine of approximately 18 g (300 mg/d for 2 months). Another paper reports a 46-year-old female patient with Sjogren’s syndrome and progressive renal disease who developed the disease after a total dose of 51 g of chloroquine [10].

It is stated that patients with a lower drug cumulative dose present faster resolution of renal and eventually systemic abnormalities, which may occur in just 6 months. In contrast, patients with numerous podocyte inclusions can have a slower clinical resolution due to the natural enzymatic difficulty in permeate these cells [18].

Given the above, it can be inferred that both chloroquine/hydroxychloroquine dose threshold required to start toxicity and the recovery time after drug withdrawal are variable, and not all patients reported in literature completely recovered from renal manifestations during follow-up. Furthermore, some cases may evolve with proteinuria improvement without total renal function recovery.

Studies tried to identify histological features in renal biopsies that would contribute to the differential diagnosis between drug-induced renal phospholipidosis and FD. Renal cell types involved by lamellar deposits in both diseases are the same and is not possible to distinguish them by morphological characteristics [19]. Typically, the most compromised cells are podocytes and endothelial cells, but smooth muscle fibers of vascular walls may also be involved [20]. In our particular case of possible drug-induced phospholipidosis, lamellar deposits were found only in podocytes while in the case of FD, they were also found in endothelial cells.

Curvilinear inclusion bodies are twisted microtubular sub-structures surrounded by a single membrane described in vascular smooth muscle cells and podocytes as a feature highly suggestive of drug-induced phospholipidosis, first reported in 2003 [10], but also typically found in cases of ceroid lipofuscinosis [21]. However, the presence of curvilinear bodies does not seem to be a uniform finding in cases of hydroxychloroquine-induced phospholipidosis [20] in agreement with our findings of no curvilinear bodies in the aforementioned patient. Thus, it is actually just a clue for differential diagnosis between FD and hydroxychloroquine-induced phospholipidosis. In addition, some studies have demonstrated the presence of lipid-laden histiocytes in the kidney, but researchers questioned whether these cells are indicative of phospholipidosis [8, 10]. However, these cells were not found in the presented cases. It was also described two other subtle ultrastructural characteristics as potential ways for differentiating iatrogenic phospholipidosis from FD. First, lamellar inclusions would be more numerous in FD and in drug-induced disease, there would be dense small, round and granular inclusions in mesangial, tubular and endothelial cells mitochondria [22]. Unfortunately, these features were not helpful to differential diagnosis in our case, as they were not found.

Treatment of hydroxychloroquine-induced phospholipidosis is mainly based in early diagnosis and drug withdrawal [23]. As in normal podocytes the activity of alpha-galactosidase is naturally low, more studies are needed to investigate the possibility of using recombinant alpha-galactosidase to help clearing lipid deposits in cases with considerable amount of podocytes inclusion bodies [9] as in our case. However, until now, there is no clinical recommendation of exogenous enzyme usage in cases of drug induced phospholipidoses, as well as there is no established dose or duration of treatment.

If Fabry disease is excluded, it means that enzyme replacement therapy, an expensive and lifelong treatment, will not be necessary. Besides, without the diagnosis of FD, the patient probably will have better life expectancy and life quality and presumably, genetic counseling will not be a concern.

## Conclusions

Drug-induced renal phospholipidosis should always be remembered as a differential diagnosis in findings on renal biopsy consistent with FD, particularly in cases with no family history or compatible clinical symptoms. A thorough report of medical records and medications in use is essential for the pathologist to consider several differential diagnoses when analyzing morphological findings in renal biopsy.

## Abbreviations

ANA: Antinuclear antibody
FD: Fabry disease
GL-3: Globotriaosylceramide
HPE2: Positive anti-nuclear antibody
SLE: Systemic lupus erythematosus
